# Tolerance of torasemide in cats with congestive heart failure: a retrospective study on 21 cases (2016–2019)

**DOI:** 10.1186/s12917-020-02554-6

**Published:** 2020-09-16

**Authors:** Camille Poissonnier, Sarra Ghazal, Peggy Passavin, Maria-Paz Alvarado, Solène Lefort, Emilie Trehiou-Sechi, Vittorio Saponaro, Alix Barbarino, Julia Delle Cave, Charlie-Rose Marchal, Boris Depré, Etienne Vannucci, Renaud Tissier, Patrick Verwaerde, Valérie Chetboul

**Affiliations:** 1 Unité de Cardiologie d’Alfort (UCA), Université Paris-Est, École Nationale Vétérinaire d’Alfort, Centre Hospitalier Universitaire Vétérinaire d’Alfort (CHUVA), 7 avenue du général de Gaulle, F-94700 Maisons-Alfort, France; 2 Pôle Anesthésie Réanimation Soins Intensifs, Université Paris-Est, École Nationale Vétérinaire d’Alfort, Centre Hospitalier Universitaire Vétérinaire d’Alfort (CHUVA), 7 avenue du général de Gaulle, F-94700 Maisons-Alfort, France; 3 U955 - IMRB Inserm, Ecole Nationale Vétérinaire d’Alfort, UPEC, F-94700 Maisons-Alfort, France

**Keywords:** Feline, Cardiomyopathy, Diuretic, Echocardiography, Pulmonary edema, Pleural effusion, Ascites

## Abstract

**Background:**

In dogs with congestive heart failure (CHF), the efficacy of torasemide, a loop diuretic, has been demonstrated. However, unlike in dogs and humans little has been described about the use of torasemide in the cat with spontaneous CHF. The objectives of this retrospective study were therefore to describe the therapeutic use of oral torasemide in cats with spontaneous CHF, document its potential adverse effects while reporting the clinical course of this feline population following torasemide administration in addition to standard medical therapy.

**Results:**

Medical records of 21 client-owned cats with CHF (median age = 10.6 years [interquartile range (IQR) = 6.5–11.2]) receiving torasemide were reviewed. Data collected included torasemide dosages, other concurrent medications, physical examination features, echocardiographic data, and potential adverse effects during follow-up. A survival analysis was performed to estimate the time from diagnosis to cardiac death. Dyspnea related to CHF was identified in all cats (pleural effusion [8/21], pulmonary edema [5/21] or both [8/21]), associated with ascites in 4/21 cats. The CHF cause was determined by echocardiography in all cats: hypertrophic (*n* = 10), restrictive (*n* = 6), arrhythmogenic right ventricular (*n* = 3), dilated (*n* = 1) cardiomyopathies, and aortic valve abnormality (*n* = 1). At initiation, median torasemide dosage was 0.21 mg/kg [IQR = 0.17–0.23] q24h. Clinical signs declined in most cats (20/21) during the first 2 weeks with no remarkable adverse events. Median survival time after torasemide prescription was 182 days [IQR = 46–330]. A contemporary control group including 54 cats with CHF, receiving furosemide as sole loop diuretic treatment was compared with the study group. Median (IQR) survival time of cats in the control group was not significatively different (*p* = 0.962) from that of the torasemide group, i.e., 148 days (9–364), although the torasemide group included significantly more cats with recurrent episodes of CHF (52%) that the control group (19%).

**Conclusions:**

This case series demonstrates that torasemide can be used in cats with spontaneous CHF. This therapeutic interest needs to be confirmed by prospective clinical trials.

## Background

In human patients and small animals with congestive heart failure (CHF), diuretics are used as the first-line treatment of clinical signs related to fluid retention (e.g., ascites, dyspnea) regardless of the underlying cause [1, 2]. Loop diuretics, such as furosemide and torasemide, act on the Na^+^:K^+^:2Cl^−^ cotransporter of the thick ascending loop of Henle, thus permitting the reduction of intravascular fluid volume with secondary decrease in preload venous and capillary pressures and relief of CHF signs [1–4]. According to the American College of Veterinary Internal Medicine (ACVIM) consensus guidelines for the diagnosis and treatment of canine myxomatous mitral valve disease (MMVD), loop diuretics are recommended for both chronic (home-based) and acute (hospital-based) treatment of MMVD dogs in ACVIM stages C and D [1].

Torasemide is an oral loop diuretic developed for human patients, which has subsequently been approved for use in dogs in Europe in 2015. Torasemide has a longer duration of action and a more potent diuretic effect than furosemide [5–8]. In experimental conditions, torasemide peak diuretic effect occurs 2 to 4 h after oral administration and this effect persists for 12 h in both dogs and cats [8]. Additionally, a recent trial on 366 MMVD dogs with past or current CHF demonstrated that torasemide administered orally once a day in addition to standard CHF therapy is non inferior to furosemide given twice a day and is associated with a two-fold reduction in the risk of reaching the composite cardiac endpoint composed of spontaneous cardiac death, euthanasia due to HF, and CHF worsening [9].

Cardiomyopathies represent the most frequent cause of CHF in the cat, and acute and chronic management of feline CHF is mainly based on oral administration of furosemide, together with the use of angiotensin-converting enzyme inhibitors (ACEI), pimobendan, and antiplatelet drugs [10–12]. However, daily oral administration of several drugs in this species is often challenging for owners and decreasing their administration frequency with long-lasting medications could improve treatment compliance.

Unlike in dogs and humans [9, 13–18], little is known about the use of torasemide in the cat with spontaneous CHF [19, 20].

The objectives of this retrospective study were therefore to 1) describe the clinical and echocardiographic features of cats with spontaneous CHF receiving oral torasemide, and 2) to determine potential adverse events secondary to oral torasemide intake in the study population and report on clinical outcome. We hypothesized that in cats with advanced heart disease, orally administered torasemide in combination with other medications would be well-tolerated.

## Results

### Patient population

The study population was composed of 21 cats with CHF treated with torasemide (16 males, 5 females; male-to-female ratio = 3.2). Median age and body weight (interquartile range (IQR); range) at inclusion (i.e., torasemide prescription) were 10.6 years (6.5–11.2; 1.8–15.4) and 4.8 kg (3.7–6.6; 3.2–7.0), respectively. The majority of cats (15/21; 71%) were domestic shorthairs, and other breeds included Persian (*n* = 1), Sphynx (*n* = 2), Norwegian (*n* = 1), Maine Coon (*n* = 1) and Chartreux (*n* = 1).

### Clinical features at the time of inclusion

All cats at inclusion presented clinical signs related to CHF.

Dyspnea secondary to pleural effusion (*n* = 8 [38%]), pulmonary edema (*n* = 5 [24%]), or both (*n* = 8 [38%]) was observed in all cats as confirmed by radiography and echocardiography, with paradoxical breathing in 5 cats (24%). Other clinical signs included abdominal distension secondary to ascites (*n* = 4 [19%]), and anorexia (*n* = 12 [57%]).

**Fig. 1:**
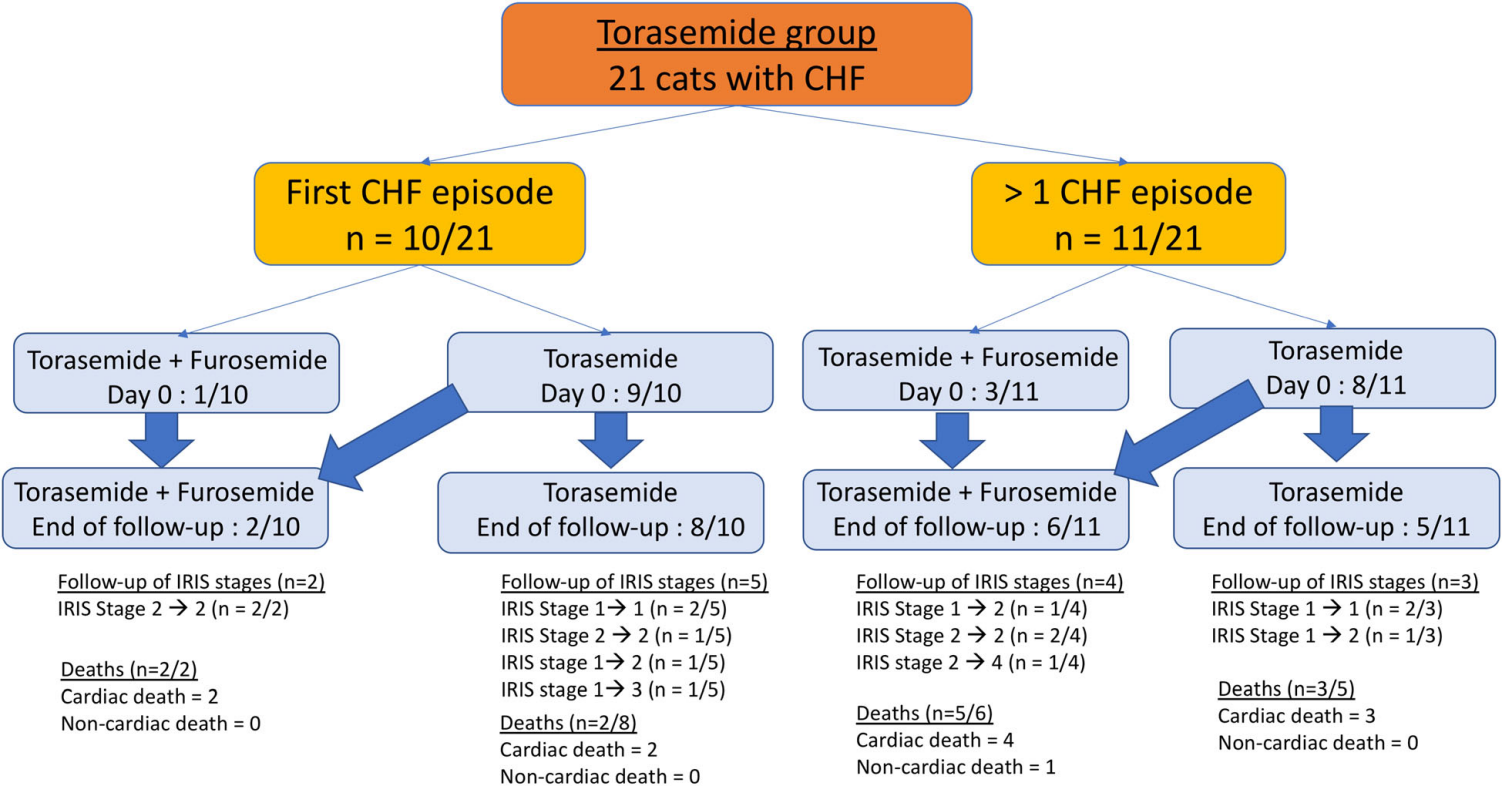
Flowchart indicating the status of cats with congestive heart failure (CHF) at initiation of torasemide prescription (*n* = 21). Among these cats, 10 cats (48%) presented with a first CHF episode requiring a first oral loop diuretic prescription and 11 (52%) had experienced at least one previous CHF episode requiring treatment adjustment owing to CHF deterioration despite furosemide prescription. Torasemide was prescribed, either alone or with an additional daily prescription of furosemide 12 h after torasemide intake (*n* = 4 at initiation of torasemide prescription, with 4 additional cats during the study period). IRIS stage evolution and death (number and causes of deaths) from Day 0 to the end of follow-up are also described for each feline group

Out of the 21 included cats, 10 (48%) presented with a first CHF episode requiring a first oral prescription of loop diuretic and 11 (52%) had experienced at least one previous episode of CHF requiring treatment adjustment owing to CHF deterioration despite furosemide prescription (Libeo, CEVA, France) once a day (*n* = 2), twice a day (*n* = 6) or three times a day (*n* = 3), and furosemide was replaced with torasemide in these 11 cats (Fig. 1). The median number of furosemide intakes was 2.0, with a median dosage [IQR; range] of 3.3 mg/kg [2.6–4.1; 1.0–5.0] q24h. Other cardiac treatments administered prior to torasemide prescription included benazepril (*n* = 7; median dosage [IQR] = 0.27 mg/kg [0.23–0.34] q24h, Fortekor, Elanco, France), clopidogrel (*n* = 5, median dosage [IQR] = 4.17 mg/kg [3.40–4.21] q24h, Plavix, Sanofi Aventis, France), aspirin (*n* = 5, 3.1 mg/kg [2.7–3.2] in one daily intake every 3 days, Aspegic Nourrisson, Sanofi Aventis, France), pimobendan (*n* = 3; median dosage = 0.18 mg/kg/day [0.15–0.32] in two daily intakes, Cardisure Eurovet Animal Health, Netherlands), spironolactone (*n* = 3; median dosage = 0.70 mg/kg [0.69–1.50] q24h, Prilactone, Ceva, France), diltiazem (*n* = 1; dosage = 3 mg/kg/day in two daily intakes, Mono Tildiem, Sanofi Aventis, France), amlodipine (n = 1; dosage = 0.13 mg/kg q24h, Amodip, Ceva, France), and taurine supplementation (*n* = 2, dosage = 100 and 110 mg/kg q24h, Felini Taurin, Futterado, Germany).

Clinical examination revealed a murmur and/or gallop sound in a total of 16/21 cats: a murmur only was detected in 7/21 cats (33%), a gallop sound only was detected in 7/21 cats (33%), and both a murmur and a gallop sound were detected in 2/21 cats (10%). The median heart murmur grade was 2/6 (range: 1 to 5/6). Among the 5/21 cats without heart murmur and/or gallop sound, auscultation revealed an irregular heart rhythm in 2/21 cats (10%), and muffled heart sounds in 1/21 cat (5%). Cardiac auscultation remained normal in 2/21 cats (10%).

Arrhythmias as confirmed by ECG tracings were detected in 5/21 cats and included atrial fibrillation (2/21), ventricular premature complexes (2/21), or both (1/21).

### Echocardiographic diagnosis at the time of inclusion

The underlying cause of CHF as determined on the basis of full echocardiographic examinations (Table 1) was hypertrophic cardiomyopathy (HCM) in 10 cats (obstructive HCM in 3 cats and nonobstructive HCM in 7 cats), restrictive cardiomyopathy in 6 cats, arrhythmogenic right ventricular cardiomyopathy in 3 cats, dilated cardiomyopathy in 1 cat, and aortic valve abnormality with secondary severe aortic regurgitation in 1 cat.

**Table 1:** Main epidemiological features as well as clinical, echocardiographic, and blood samples variables assessed at inclusion in the study group (*n* = 21 cats with congestive heart failure treated with torasemide) as compared with a contemporary control group of 54 cats with congestive heart failure receiving furosemide as sole loop diuretic treatment

	Population of 21 cats treated with torasemide			Control Group (n = 54)		
	n	Median (1^st^ quartile - 3^rd^ quartile)	Minimum - Maximum	n	Median (1^st^ quartile – 3^rd^ quartile)	Minimum - Maximum
**Epidemiological features**						
Age (years)	21	10.6 (6.5–11.2)	1.8–15.4	54	7.2 (5.6–9.8)	0.9–18.2
Weight (kg)	21	4.8 (3.7–6.6)	3.2–7.0	54	5.3 (4.5–6.3)	3.3–7.3
Sex (M/F; %)	16/5; 76/24	–	–	33/21		–
**Clinical data**						
Heart Murmur (yes/no; %)	9/12; 43/57	–	–	26/28; 48/52	–	–
Gallop sound (yes/no; %)	9/12; 43/57	–	–	7/47; 13/87	–	–
Arrhythmia (yes/no, %)	5/16; 24/76	–	–	0/54; 0/100	–	–
Minimal heart rate	21	225 (192–245)	170–280	54	200 (177–230)	120–320
**Pre-treatment**						
Furosemide (yes/no; %)	11/10; 52/48	–	–	37/17; 69/31	–	–
Benazepril (yes/no; %)	7/14; 33/67	–	–	15/39; 28/72	–	–
Clopidogrel (yes/no; %)	5/16; 24/76	–	–	16/38; 30/70	–	–
Spironolactone (yes/no %)	3/18; 14/86	–	–	2/52; 4/96	–	–
Pimobendan (yes/no; %)	3/18; 14/86	–	–	3/51; 6/94	–	–
Taurine (yes/no; %)	2/19; 10/90	–	–	2/52; 4/96	–	–
Amlodipine (yes/no; %)	1/20; 5/95	–	–	0/54; 0/100	–	–
Diltiazem (yes/no; %)	1/20; 5/95	–	–	0/54; 0/100	–	–
Atenolol (yes/no; %)	0/21; 0/100	-	-	1/53; 2/98	-	-
**Echocardiography**						
Pleural effusion identified (yes/no; %)	16/5; 76/24	–	–	24/30	–	–
LA:Ao ratio at end-diastole	21	1.99 (1.82–2.25)	1.28–3.50	54	1.88 (1.61–2.10)	1.30–2.70
LA dilation (yes/no; %)	21/0;100/0	–	–	54/0; 100/0	–	–
RA (mm)	21	14 (12–20)	9.7–21.1	54	14.0 (11.5–15.5)	6.4–24.8
RA dilation (yes/no; %)	10/11; 48/52	–	–	17/37; 31/69	–	–
FS (%)	21	42 (31–49)	22–69	54	41 (36–49)	15–75
**Blood sample**						
Urea (g/L)	18	0.67 (0.56–0.76)	0.44–1.19	45	0.67 (0.56–0.82)	0.40–1.99
Creatinine (mg/L)	18	15.4 (12.2–17.4)	9.0–27.0	47	14.1 (10.7–20.7)	5.0–38.0
Potassium (mmol/L)	13	3.7 (3.5–4.4)	3.0–5.3	41	4.0 (3.4–4.6)	2.6–5.3
Sodium (mmol/L)	13	151 (150–157)	143.0–163.0	36	152 (147–157)	137–174
IRIS Stage (n; stages 1/2/3; %)	18; 10/8/0; 56/44/0	–	–	47; 27/18/2; 57/38/4	–	–

*F female; FS fractional shortening; IRIS International Renal Interest Society; LA left atrium; LA:Ao left atrium to aorta ratio; M male; RA right atrium*

The left atrium (LA) to aorta (Ao) ratio (LA:Ao) was increased in all cats with a median end-diastolic LA:Ao ratio of 1.99 (IQR = 1.82–2.25; normal values ≤1.2 [21]). Left atrial dilation was considered as severe (i.e. end-diastolic LA:Ao ratio ≥ 2.0 [22]) in 12/21 (57%) cats. The median end-diastolic RA diameter was 14 mm (IQR = 12–20; normal values ≤15 mm), with a right atrial (RA) dilation found in 10/21 (48%) cats [22] (Table 1).

### Serum chemistry values and IRIS stages at the time of inclusion

A blood sample was obtained in 18/21 cats before torasemide initiation: median (IQR) creatinine and urea values were 15.4 mg/L (12.2–17.4; reference ranges = 5.2–17.8) and 0.67 g/L (0.56–0.76; reference ranges = 0.40–0.80), respectively, and median (IQR) serum potassium was 3.7 mmol/L (3.5–4.4; reference ranges = 3.6–5.5, Table 1). According to the International Renal Interest Society (IRIS) classification, 10 cats were in IRIS stage 1 and 8 were in IRIS stage 2. For the 3 remaining cats, blood could not be sampled because of severe dyspnea and/or marked stress.

### Treatment

The median torasemide dosage at initiation was 0.21 mg/kg [IQR = 0.17–0.23; range = 0.11–0.35] q24h. For 17 cats, a single oral intake of loop diuretic (i.e., torasemide) was prescribed (as a first-line diuretic for 9/17 cats or as replacement of furosemide for 8/17 cats) whereas for 4 other cats another daily oral intake of loop diuretic (i.e., furosemide) was needed 12 h after torasemide administration owing to severe signs of CHF (median dosage of furosemide [IQR; range] = 1.63 mg/kg [1.44–1.83; 1.09–2.20] q24h). Among those 4 cats, 3 had experienced a previous episode of CHF and were already receiving furosemide before torasemide initiation (Fig. 1), and in one cat furosemide was administered in addition to torasemide as a first-line treatment. Concurrent medical therapy was adjusted for all cats, and included benazepril for 5 cats (median dosage [range] = 0.27 mg/kg [0.25–0.28] q24h, those 5 cats were already receiving benazepril prior to torasemide initiation), pimobendan for 6 cats (median dosage = 0.37 mg/kg/day [0.30–0.44] in two daily intakes, including the 3 cats already receiving pimobendan prior to torasemide initiation), and spironolactone for 4 cats (median dosage = 0.73 mg/kg [0.65–1.15] q24h, including the 3 cats already receiving spironolactone prior to torasemide initiation). Antithrombotic therapy was also administered to most cats (19/21, 90%) and included clopidogrel (16 cats, median dosage [IQR] = 3.56 mg/kg [2.83–4.25] q24h, including the 5 cats already receiving clopidogrel prior to torasemide initiation) or aspirin (3 cats, median dosage [IQR] = 3.13 mg/kg [2.99–3.13] in one intake every 3 days, those 3 cats were already receiving aspirin prior to torasemide initiation).

### Follow-up

Follow-up data was available for all cats included the study. Median follow-up time for all cats was 85 days (IQR = 40–143; range = 2–347). Torasemide dosage was increased for 33% (7/21) cats owing to the persistent CHF signs (i.e., persistence of dyspnea at clinical examination, with persistence of pulmonary edema and/or pleural effusion (thoracic radiographs)) at one of the follow-up visits at a median time of 7 days after initiation (minimum-maximum = 2–140) (median dosage [IQR; range] = 0.22 mg/kg [0.17–0.31; 0.08–0.40] q24h), and torasemide dosage was decreased for 1/21 cat (5%) 15 days after initiation. For 4 out of the 17 cats initially receiving only a single oral intake of loop diuretic (i.e., torasemide), furosemide was added daily 12 h after torasemide intake, at a median time of 39 days after torasemide initiation (minimum-maximum = 7–291). Thus, a total of 8/21 cats (38%) were prescribed furosemide in addition to torasemide (median [IQR; range] furosemide dosage at the end of follow-up = 1.29 mg/kg [1.09–1.83; 0.50–2.34] q24h; median [IQR, range] torasemide dosage at the end of follow-up = 0.29 mg/kg [0.25–0.36; 0.16–0.40] q24h). Among those 8 cats, 6 had experienced a previous episode of CHF and had already received furosemide before torasemide initiation. For the 11 cats that had experienced at least one previous episode of CHF and were previously treated with furosemide, the median [range] number of daily loop diuretic intakes was decreased from 2.0 [1.0–3.0] to 1.0 [1.0–2.0].

Regression of clinical signs with marked improvement of general clinical condition and no overt adverse events were observed in all cats during the first 2 weeks following initiation except one (20/21) that died 48 h after treatment initiation (sudden death). This death was considered as cardiac-related death since the animal died suddenly while concomitantly suffering from severe CHF without any overt signs of noncardiac diseases.

**Fig. 2:**
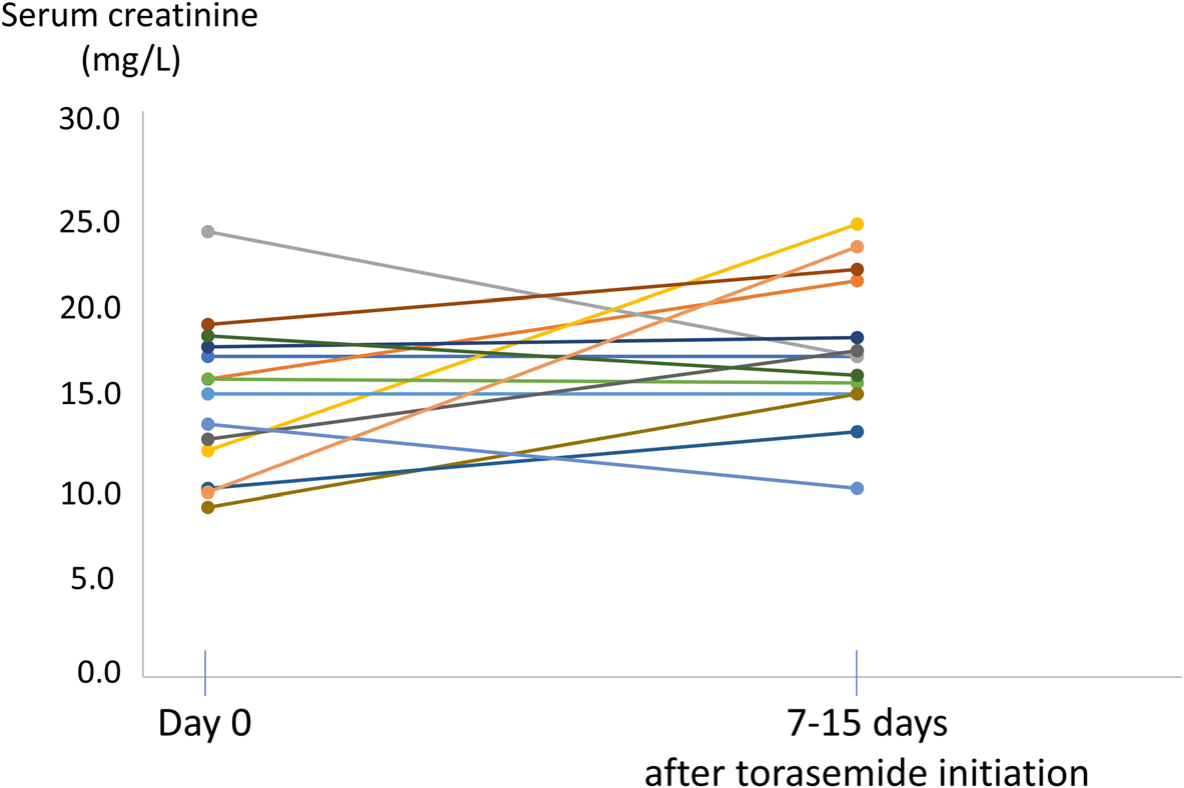
Serum creatinine evolution after torasemide initiation. Each plot represents serum creatinine values before and 7 to 15 days after torasemide initiation, for the 14 cats with an available creatinine follow-up (reference ranges = 5.2–17.8 mg/L). Median [IQR] creatinine values (15.4 mg/L [12.2–17.4] and 17.0 mg/L [15.2–20.3] before and after torasemide initiation, respectively) were not significantly different (*p* = 0.12)

During the 2 weeks following torasemide initiation, 5 out of the 14 torasemide cats with an available blood sample follow- up remained in IRIS stage 1, 2/14 turned from IRIS stage 1 to stage 2, and 7/14 cats remained in IRIS stage 2. IRIS stages were not significantly different before and after the treatment initiation (*p* = 0.45). Median (IQR) serum creatinine values after the 2 first weeks of treatment were 17.0 mg/L [15.2–20.3] and were not significantly different from initial values (15.4 mg/L [12.2–17.4]; *p* = 0.12) (Fig. 2). During the same period, follow-up electrolyte levels were available for 12 cats. Potassium serum concentration remained within reference ranges for 10/12 cats and was decreased for only 2 cats (i.e. 3.3 mmol/L and 3.2 mmol/L, reference ranges = 3.6–5.5 mmol/L), thus requiring potassium supplementation (potassium gluconate).

In addition, for the 10/14 torasemide cats with a creatinine follow-up of more than 2 weeks, median (IQR) serum creatinine values at the last blood sampling were not significantly different from the initial values (*p* = 0.12). Follow-up creatinine values were available for 7/10 cats for periods of less than 60 days (median [IQR] duration = 32 [18–40] days), and creatinine remained < 28 mg/L (IRIS stages 1 and 2) except for one cat (median [IQR] = 18.5 mg/L [14.8–23.3]). Of the remaining three cats with a longer creatinine follow-up (182, 183 and 252 days), IRIS stage did not change for two cats (creatinine of 17 mg/L versus 17 mg/L at initiation for one cat and creatinine of 19 mg/L versus 15 mg/L for the second one), whereas it increased for one cat which experienced acute renal failure (serum creatinine and urea values of 56.9 mg/L (versus 23,6 mg/L) and 2.34 g/L *(*versus 0.76 g/L, respectively). This cat experienced a recurrence of acute pulmonary edema following a marked stressful episode (grooming by the owner) 167 days after torasemide initiation, and subcutaneous injections of furosemide were used for 1 week as replacement of the oral furosemide while torasemide dosage remained unchanged. Injections were then stopped and replaced with oral furosemide at a higher dosage than initially prescribed (1.0 mg/kg q24h versus 0.42 mg/kg q24h). Euthanasia was elected 2 weeks later (i.e., 182 days after torasemide initiation). Overall, the IRIS stages of the 10 cats with available follow-up values over 2 weeks were not significantly different at the end of the study as compared to initial stages (*p* = 0.48), although IRIS stage increased for 4/10 cats at the end of the study as compared to IRIS stages before torasemide initiation. Among those 4 cats, 3/4 received torasemide only, and 1/4 also received furosemide in addition to torasemide.

**Fig. 3:**
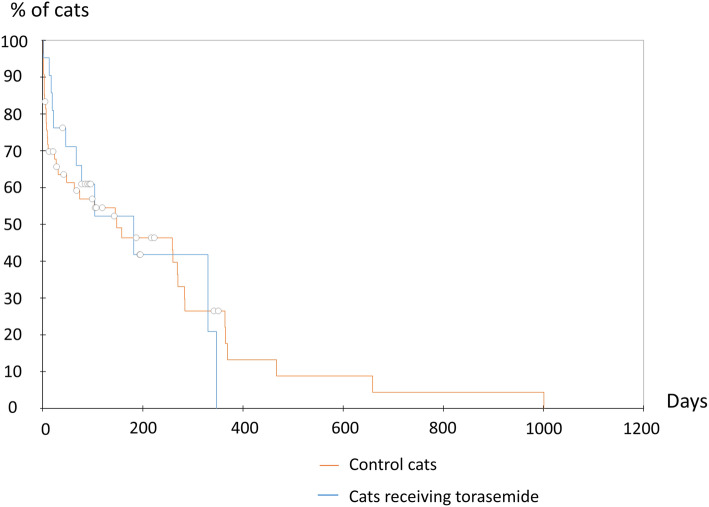
Kaplan-Meier curves of survival time from initial torasemide prescription to all-cause death, compared with the control group. Median (IQR; min-max) survival time from diagnosis was 182 days (46–330; 2–347) for the 21 included cats receiving torasemide (with or without furosemide), and 148 days (9–364, 2–1001) for the control group (*n* = 54 cats receiving furosemide as sole loop diuretic treatment). Median survival time of cats from the control group was not significatively different from that of the torasemide group, *p* = 0.962. Circles denote censored observations

At the end of the study (September 2019), death was reported in 12/21 cats from the torasemide group including one renal death (8%) and 11 cardiac deaths (92%), i.e., euthanasia for 6/11 cats owing to CHF worsening (i.e., pulmonary edema) despite ongoing treatments (*n* = 3) or aortic thromboembolism (*n* = 3) and spontaneous cardiac deaths for 5/11 cats related to CHF worsening (i.e., acute pulmonary edema leading to death despite ongoing treatments; n = 3) or sudden cardiac arrest (*n* = 2). Median survival time (all-cause death) was 182 days [IQR = 46–330; range = 2–347] following torasemide prescription (Fig. 3).

A contemporary control group of cats with CHF for which furosemide was the sole loop diuretic prescribed during the same study period was retrospectively recruited for comparison with the group of cats receiving torasemide. During the study period, a total of 408 cats that did not receive torasemide were diagnosed with heart diseases, among which 109 had CHF. Among these 109 CHF cats, 23 cats were excluded from the control group owing to a systemic concomitant disease (i.e., hyperthyroidism, systemic arterial hypertension, diabetes mellitus, cancer, hepatitis, pancreatitis) and 32 cats were excluded owing to a transient myocardial thickening during follow-up (*n* = 5) or a follow-up < 24 h (*n* = 27). The selected control group included therefore 54 cats of 9 different breeds with a median (IQR) age of 7.2 years (5.6–9.8) and body weight of 5.3 kg (4.5–6.3). The included breeds were the following: Domestic shorthair cats (*n* = 37, 69%), Chartreux (*n* = 4, 8%), Maine Coon (*n* = 3, 5%), Birman (*n* = 3, 5%) Sphynx (*n* = 3, 5%) Bengal (*n* = 1, 2%), British Shorthair (*n* = 1, 2%), Norwegian Forest cat (*n* = 1, 2%) and Persian cat (*n* = 1, 2%). Cardiac diseases causing CHF in these control cats included HCM (29/54, 54%), restrictive cardiomyopathy (12/54, 22%), dilated cardiomyopathy (3/54, 5%), arrhythmogenic right ventricular cardiomyopathy (2/54, 4%), and nonspecific cardiomyopathy (8/54, 15%) [23]. Among these 54 control cats, a blood sample was available for 47 (87%): 27/47 (57%) were classified as IRIS stage 1, 18/47 (38%) as IRIS stage 2, and 2/47 (4%) as IRIS stage 3 with median (IQR) serum creatinine values of 14.1 mg/L (10.7–20.7), which were not significantly different from that of the torasemide group (*p* = 0.79). The LA:Ao was increased in all control cats with a median end-diastolic LA:Ao ratio of 1.88 (IQR = 1.61–2.10). Left atrial dilation was considered as severe (i.e., end-diastolic LA:Ao ratio ≥ 2.0 [22]) in 20/54 (37%) cats. The median end-diastolic RA diameter was 14.0 mm (IQR = 11.5–15.5), with a RA dilation found in 17/54 (31%) cats [22]. Age (*p* = 0.053), body weight (*p* = 0.650), creatinine values (*p* = 0.790), LA:Ao ratio (*p* = 0.079), RA diameter (*p* = 0.070) were not significantly different between the two groups.

Out of the 54 included control cats, 44 (81%) presented with a first CHF episode requiring a first oral prescription of loop diuretic while 10 (19%) had experienced at least one previous episode of CHF requiring treatment adjustment owing to CHF deterioration despite furosemide prescription. This was significatively different from the torasemide group in which only 10/21 (48%) cats presented with a first CHF episode requiring an initial loop diuretic prescription (*p* = 0.008).

Median (IQR, min-max) survival time (all-cause death) of cats from the control group was not significatively different from that of the torasemide group, i.e., 148 days (9–364, 2–1001), *p* = 0.962 (Fig. 3). For control cats with an available blood sample follow-up (*n* = 25), the median (IQR, range) time for creatinine follow-up was 18 days (8–75, 2–340), which was not significatively different from that of the torasemide group (*p* = 0.87). Out of these 25 cats, 5/25 remained in IRIS stage 1, 6/25 turned from IRIS stage 1 to stage 2, 7/25 cats remained in IRIS stage 2, 2/25 turned from IRIS stage 2 to stage 1, 3/25 turned from IRIS stade 2 to stage 3, 1/25 turned from IRIS stage 3 to stage 2, and 1/25 turned from IRIS stage 3 to stage 1. No significant differences (*p* = 0.68) regarding creatinine values were found at the end of follow-up between control cats (receiving furosemide as sole loop diuretic treatment, *n* = 25) and cats treated with torasemide with or without furosemide (*n* = 14).

## Discussion

Torasemide has been recently described as a new loop diuretic for the treatment of spontaneous canine congestive heart failure [9] and approved for use in Europe. The present retrospective study provides original data regarding the practical use of torasemide in a series of cats with spontaneous CHF. In the dog, torasemide half-life (12 h) is twice as long as that of furosemide (6 h), its bioavailability is also higher (80 to 100% versus maximum 77%), with a 10 to 20 fold more potent diuretic effect [5, 8, 24, 25]. Therefore, the number of administrations can be reduced from three to four times a day for furosemide to once to twice a day for torasemide in dogs. This property would be particularly interesting in the feline species, in which daily drug administration is often challenging for owners. However, to the best of our knowledge, no prospective clinical trial has yet evaluated the use of torasemide in spontaneous feline CHF. Nevertheless, Uechi et al. suggested that torasemide is useful for treating CHF in cats, with a 1:10 equivalence between torasemide and furosemide [8]. This series of 21 cases confirms that torasemide can reduce congestion in cats with CHF in both first-line and second-line prescriptions with a median survival time of six months, and up to nearly one year for one cat. Additionally, for cats already under furosemide treatment at inclusion (owing to at least one previous episode of CHF), the median number of daily administrations of loop diuretics was decreased from 2.0 to 1.0 daily oral intakes after torasemide initiation. In the present series, median survival time was longer than that in two previous descriptions (median [min-max] survival time of 182 days [2–347] with a median dosage at the end of the study = 0.22 mg/kg/day [0.17–0.31] versus 87 days [3–466] and 48 [7–177]) [19, 20]. However, in both studies, the type of heart disease (for the respectively 17 and 11 included CHF cats) was not available and such differences in survival could be related to differences in the underlying cardiac diseases presented. The cats of the current study had various heart diseases, which might differ from cats represented in the previously cited abstracts.

Additionally, in the present report, more than one third of included cats required a second administration of loop diuretic (furosemide) 12 h after torasemide oral intake, which may have influenced the present results to an unknown extent.

Except for one sudden death 48 h after torasemide initiation, no clinical adverse effect was reported in the 21 included cats and no worsening of IRIS stages was observed within the first 2 weeks. Only 2 cats showed a mild decrease in potassium serum concentration below reference ranges leading to potassium supplementation. During the long-term follow-up, serum creatinine values were not significatively different as compared to inclusion values and only one cat underwent acute renal failure 6 months after torasemide initiation and 2 weeks after furosemide increase owing to acute recurrent pulmonary edema. The potential respective contribution of torasemide and furosemide to this renal failure occurrence is currently uncertain, and this uncertainty represents one of the limitations of the present series. Cats are known to be predisposed to renal failure, and development or worsening of preexistent acute or chronic renal failure in cats with CHF is a commonly observed comorbidity [26]. Nevertheless, no significant differences regarding serum creatinine values were found between control cats (receiving furosemide as sole loop diuretic treatment) and cats treated with torasemide with or without furosemide at the end of follow-up.

This case series has several limitations, mainly related to its retrospective nature and the small sample size. Values for several biochemical variables were unavailable for few animals and medical treatments associated with torasemide were not standardized. Additionally, included cats belonged to different breeds and suffered from various heart diseases, which could have influenced the present data and torasemide tolerance to an unknown extent. Owing to financial constraints no abdominal ultrasound was performed in the cat with acute renal failure whose precise cause remains unknown. Finally, when cats did not die in our hospital, the cause of death (cardiac versus noncardiac) was determined based on the descriptions of the veterinarians and owners, which could therefore lead to misclassification and this represents another limitation of the study. In the present study, the torasemide group included significantly more cats with recurrent episodes of CHF (52%) that the control group (19%), suggesting more advanced heart diseases for the former, which could have influenced the results of survival times to an unknown extent.

## Conclusions

This case series illustrates that torasemide can be used in cats with spontaneous CHF as first-line and second-line loop diuretic treatment, with a good general tolerance and very few adverse effects, during the available follow-up. Further prospective case-controlled clinical trials with larger numbers of cats are now needed to confirm the long-term safety and efficacy of torasemide as compared to furosemide in feline CHF.

## Methods

### Study design

This study was carried out as a retrospective study.

### Study population

The study population was composed of client-owned cats with CHF secondary to naturally occurring heart disease diagnosed at the Alfort Cardiology Unit (National Veterinary School of Alfort, France) between September 2016 and May 2019. Animals with equivocal echocardiographic and/or radiographic findings were not included in the study. Owners’ consent for off-label torasemide use was obtained.

### Medical records review

Electronic medical records of all the included animals were carefully evaluated. Collected data included signalment (age, sex, body weight), clinical signs at the time of CHF diagnosis, type of cardiac disease diagnosed by echocardiography, blood parameters (urea, creatinine, sodium, potassium), IRIS stages, therapy at the time of diagnosis, and outcome of the recruited animals. In all patients, CHF was defined by the presence of pulmonary edema and/or pleural, pericardial, and abdominal effusions confirmed by radiography, echocardiography and/or abdominal ultrasound examination. Radiographs were performed on two different views (lateral and ventral views) to confirm the presence of CHF. Torasemide dosages and administration frequency were recorded, as well as concurrently administered cardiac and noncardiac medications. Response to treatment within the first 15 days was evaluated by clinical examination performed by the same veterinarian who did the initial clinical and echocardiographic examinations. In case of pulmonary edema and/or pleural effusion, resolution of CHF was defined as disappearance of both CHF-related clinical signs (e.g., dyspnea) and radiographic signs of CHF. Additionally, the following questions were asked to the owners at inclusion and during the follow-up: presence or absence of clinical signs potentially related to heart diseases (dyspnea, cough, exercise tolerance, syncope, others), presence or absence of other clinical signs (decreased appetite, vomiting, diarrhea, others) related to potential adverse events of the ongoing treatment, and treatment compliance (drugs, dosages, and time of last administration). Owners were contacted by phone or email at the end of the study to enquire about any other potential adverse events, which were also registered (date and description).

### Echocardiographic data

Standard transthoracic M-mode, 2-dimensional (2D), and Doppler examinations with concomitant ECG tracings, if possible, were performed by trained observers (Dipl. ECVIM-CA Cardiology, final-year resident, clinician with at least 4 years of experience at our unit) in awake cats by the use of ultrasound units^1^ equipped with phased-array transducers, as previously described and validated [27]. Echocardiographic examinations performed by less trained observers were not included in the study. Data from the original echocardiographic reports were collected. Additionally, all images were reviewed off-line by a board-certified cardiologist (VC), who was blinded to the identity and the clinical status of the animal.

Recorded echocardiographic data included the presence or absence of pleural and pericardial effusion. The type and severity of heart diseases was based for all cats on the following measurements [10]: left ventricular (LV) end-diastolic and end-systolic internal diameters, LV free wall thicknesses at end-diastole and end-systole, interventricular septum (IVS) thicknesses at end-diastole and end-systole measured by the use of 2D-guided M-mode [28, 29], the corresponding LV shortening fraction, as well as the end-diastolic LA:Ao ratio and the end-diastolic RA diameter, respectively measured from the 2D right parasternal transaortic short axis view and 4-chamber view, as previously described [22, 27, 30]. The LA was considered as severely dilated for LA:Ao values ≥2 [22]. The end-diastolic RA was judged as dilated if its diameter (measured at the level of the tricuspid annulus from the 2D right parasternal 4-chamber view) was wider than the upper reference limit (i.e., > 15 mm) obtained from a population of 120 healthy adult cats [22]. The sub-aortic IVS thickness was also measured at end-diastole using 2D mode from the right parasternal 5-chamber view at the level of the attachments of the *chordae tendineae* to the mitral valve leaflets [30]. Pulsed-wave and continuous-wave Doppler modes were used to record blood flow velocities. Maximum early (E) and late (A) diastolic mitral flow velocities were determined using pulsed-wave Doppler mode from the left apical 4-chamber view, and the mitral E:A ratio then was calculated. Continuous-wave Doppler recorded from the left apical 5-chamber view was used to confirm or exclude LV outflow tract obstruction characterized by turbulent aortic flow of high velocity (> 2 m/s) [21]. The presence or absence of systolic anterior motion of the mitral valve also was evaluated using both 2D and M-modes [31]. Finally, color-flow and spectral Doppler modes were used to identify potential valve regurgitations, shunting and stenotic lesions.

### Electrocardiography

All cats with suspected arrhythmias on cardiac auscultation underwent a 6-lead ECG examination^2^ (I, II, III, aVL, aVR, aVF) during 5 min, in addition to concomitant ECG tracings performed during echocardiographic examinations.

### Follow-up

Owners of cats for which the outcome was unknown in the database at the end of the study (September 2019) were contacted by telephone or e-mail to determine the current status of their animal: alive or dead (date and cause of death, if known). Cause of death was defined as cardiac or noncardiac, according to the information provided by the owners or the evaluation of veterinarians when the animal died during hospitalization. Cardiac death was defined as death following worsening of CHF despite ongoing treatments, sudden death without an obvious noncardiac cause, or euthanasia owing to worsening of CHF signs such as ascites, pulmonary edema, and pleural effusion. If none of the criteria for cardiac-related death were fulfilled, cause of death was qualified as noncardiac. Owners were also asked about the occurrence of potential adverse effects: the date and details about the exact clinical signs were recorded. Animals for which the outcome could not be obtained at the time of writing (September 2019) were censored at the date of their last examination. Change in torasemide dosages during follow-up was also noted.

A control group of cats with CHF for which furosemide was the sole loop diuretic prescribed during the same study period was composed in order to compare survival with cats treated with torasemide. The inclusion criteria for this contemporary control group were the presence of CHF as confirmed by thoracic radiographs (pulmonary edema or pleural effusion) and/or echocardiography (pleural or pericardial effusion), echocardiographic examinations performed by the same above-mentioned trained observers, and a follow-up period > 24 h. Cats with concomitant diseases (e.g., hyperthyroidism, systemic hypertension, diabetes) and with identification of transient myocardial thickening during follow-up were not included in the control group.

### Statistical analysis

Commercially available software (Addinsoft (2019); XLSTAT statistical and data analysis solution; USA) was used for statistical analyses. The normality of variables was confirmed by applying Kolmogorov-Smirnov analysis, for creatinine values before and within the first 2 weeks after torasemide initiation. Data were expressed as proportions or percentages (%), medians, IQR, and minimum-maximum values and were compared using paired Student t-test (for creatinine values before and after torasemide initiation), and Mann-Whitney non-parametric analysis (for variables between torasemide and control groups and for IRIS stages before and after torasemide initiation). Median survival time to all-cause death was estimated among the two groups using the Kaplan-Meier method and compared using the log rank test.

## Data Availability

The datasets used and analyzed during the current study are available from the corresponding author on reasonable request.

## Abbreviations

2D: Two-dimensional
ACVIM: American College of Veterinary Internal Medicine
ACEI: Angiotensin-converting enzyme inhibitors
Ao: Aorta
CHF: Congestive heart failure
MMVD: Myxomatous mitral valve disease
HCM: Hypertrophic cardiomyopathy
IQR: Interquartile range
IRIS: International Renal Interest Society
IVS: Interventricular septum
LA: Left atrium
LV: Left ventricular
RA: Right atrium

## Footnotes

1 Vivid 7, Vivid E9; General Electric Healthcare; USA.

2 MAC 5500 HD ECG Analysis System; General Electric Healthcare; USA.
